# The College of Nursing: A Glasgow View

**Published:** 1916-09-23

**Authors:** 


### THE EDITOR'S LETTER-BOX.

### The College of Nursing: A Glasgow View.

*To the Editor of* THE HOSPITAL.

SIR,—As one specially interested in the training of nurses and the welfare of the nursing profession, I would crave your indulgence while I pass some remarks on the first local general and public meeting of the recently instituted College of Nursing, held last week in Glasgow.

Until the present, all the information which the general medical and nursing public have received on the matter has been obtained from occasional newspaper paragraphs stating how a committee had been considering the best means of improving the status of the nurse, safeguarding her interest and also that of the general public, with the result that a College of Nursing had been established as a limited company, and that it was the intention of this body to approach Parliament with a Registration Bill. Details of the scheme have always been wanting, and consequently I, as no doubt many others, felt distinctly disappointed with the result of the meeting.

We were told that the College of Nursing was an established fact, that it had, as a limited company, received the sanction of the Board of Trade, and that within a month the Committee hoped to conduct a Registration Bill through Parliament, and that there was every hope of success, as the nursing and medical professions were unanimous in their opinion of the scheme. The Hon. Mr. Stanley (who is the Chairman of the Council of the College of Nursing) spoke at length on the above points, but not a word was said as to how it was intended to bring to fruition the laudable objects for which the College was founded. Absolutely no details were given of a scheme which has such a far-reaching effect on the nursing profession, and which they, as nurses, are asked to support.

Surely, if the Bill is to be laid before Parliament within a month, the Council must have some idea of their plans, and, what is more important, the nurses ought to be informed of the manner in which their interests are to be safeguarded—the safeguarding of the nurses' interests was a great point of Mr. Stanley's speech—but on this there was not the slightest attempt to enlighten. Nevertheless, the nurses were asked to send in their names in support of the scheme, although appended to the application form for registration was a small notice asking the applicant not to forward the registration fee of £1 1s. until she had been notified by the Council that her application had been accepted, and no indication was given of the training and experience on which acceptance or non-acceptance would be based. In short, the nurse was asked to sign a blank cheque and to record her vote of confidence, not only in a scheme of which she knew nothing and which might modify her future career materially, but also in men and women who simply said they would look after her interests. The Council was very anxious, said Mr. Stanley, to settle all differences outside Parliament, but I hardly think that the meeting held in Glasgow would help much towards that end.

During the course of his remarks the Hon. Mr. Stanley mentioned one very important fact, viz. the fate of fever and children's nurses. He remarked that he had often been asked what would be their position under the scheme, and he simply said that he could not tell, but that a matter of two years would show—they were to wait and see, but in the meantime the College of Nursing and the Registration Bill would have received the Royal Assent.

Questions were called for, but the only one of importance was forestalled by Mr. Stanley in his speech, viz. the position of fever and children's nurses. Hence there were no questions, and in his reply Mr. Stanley remarked on the unanimity with which the scheme seemed to be viewed by the nurses of Glasgow, a state of matters which, he said, was similar to what prevailed in Manchester.

Regarding the relative value of training in different institutions I do not intend to speak, but that those nurses—viz. fever and children's nurses—who probably see more acute disease, and at a period of life most difficult to nurse, than the so-called general nurse, are to be left outside and to take a back seat in the nursing profession is a very serious matter. I have not absolute figures to quote, but, so far as Glasgow is concerned, there are probably more nurses being trained in fever, parochial, and children's hospitals than in the general infirmaries, and any scheme which will not include these women and give them their due position can hardly be expected to receive the unanimous support of the nursing profession.—I am, etc., ONE INTERESTED.

Glasgow, September 18.

[This temperate letter raises, in effect, two distinct issues: a general criticism of the methods of the promoters of the College of Nursing, and a special one of the position of nurses trained at children's and fever hospitals. With regard to the first one, it is doubtless true that the daily press has thrown little light on the plans and policy of Mr. Stanley and the College of Nursing; but THE HOSPITAL, and to a lesser degree the principal nursing papers, have given very full and frequent information for months past on precisely these points. In regard to one, that of the training and experience to qualify for admission to the Register, this information was given in full in THE HOSPITAL of June 10, 1916, p. 237. With regard to our correspondent's other issue, the status of fever and children's hospital nurses is a complicated matter about which any hasty decision would be certain to cause controversy. In postponing a solution of this particular problem for the consideration of the Consultative Board (see Mr. Stanley's statement at the College of Nursing meeting of June 15, THE HOSPITAL, June 24, p. 313) Mr. Stanley is taking a prudent course; and we would point out to our correspondent that the peculiar advantage of this postponement is that the whole matter can then be settled by nurses themselves through their representatives, after full and expert consideration of all sides of a somewhat thorny question.—ED. THE HOSPITAL.]

